# Effects of replacing PSA with Stockholm3 for diagnosis of clinically significant prostate cancer in a healthcare system – the Stavanger experience

**DOI:** 10.1080/02813432.2020.1802139

**Published:** 2020-08-08

**Authors:** Eirik Viste, Cathrine Alvaer Vinje, Torgeir Gilje Lid, Svein Skeie, Øystein Evjen-Olsen, Tobias Nordström, Olav Thorsen, Bjørnar Gilje, Emiel A. M. Janssen, Svein R. Kjosavik

**Affiliations:** a Faculty of Health Sciences, University of Stavanger, Stavanger, Norway; b The General Practice and Care Coordination Research Group, Stavanger University Hospital, Stavanger, Norway; c Department of Urology, Stavanger University Hospital, Stavanger, Norway; d Department of Clinical Science, University of Bergen, Bergen, Norway; e Department of Research, Stavanger University Hospital, Stavanger, Norway; f Organization and Development Unit SUS 2023, Stavanger University Hospital, Stavanger, Norway; g Department of Medical Epidemiology and Biostatistics, Karolinska Institutet, Stockholm, Sweden; h Department of Oncology, Stavanger University Hospital, Stavanger, Norway; i Department of Pathology, Stavanger University Hospital, Stavanger, Norway; j Faculty of Science and Technology, University of Stavanger, Stavanger, Norway

**Keywords:** Diagnostic methods, family medicine, Gleason score, health economy, implementation study, over diagnosis, prostate cancer, PSA, Stockholm3

## Abstract

**Objective:**

To describe early experience of replacing PSA with Stockholm3 for detection of prostate cancer in primary care.

**Design and methods:**

Longitudinal observations, comparing outcome measures before and after the implementation of Stockholm3.

**Setting:**

Stavanger region in Norway with about 370,000 inhabitants, 304 general practitioners (GPs) in 97 primary care clinics, and one hospital.

**Intervention:**

GPs were instructed to use Stockholm3 instead of PSA as standard procedure for diagnosis of prostate cancer.

**Main outcome measures:**

Proportion of GP clinics that had ordered a Stockholm3 test. Number of men referred to needle biopsy. Distribution of *clinically significant prostate cancer* (csPC) (Gleason Score ≥7) and *clinically non-significant prostate cancer* (cnsPC) (Gleason Score 6), in needle biopsies. Estimation of direct healthcare costs.

**Results:**

Stockholm3 was rapidly implemented as 91% (88/97) of the clinics started to use the test within 14 weeks. After including 4784 tested men, the percentage who would have been referred for prostate needle biopsy was 29.0% (1387/4784) if based on PSA level ≥3ng/ml, and 20.8% (995/4784) if based on Stockholm3 Risk Score (*p* < 0.000001). The proportion of positive biopsies with csPC increased from 42% (98/233) before to 65% (185/285) after the implementation. Correspondingly, the proportion of cnsPC decreased from 58% (135/233) before to 35% (100/285) after the implementation (*p* < 0.0017). Direct healthcare costs were estimated to be reduced by 23–28% per tested man.

**Conclusion:**

Replacing PSA with Stockholm3 for early detection of prostate cancer in primary care is feasible. Implementation of Stockholm3 resulted in reduced number of referrals for needle-biopsy and a higher proportion of clinically significant prostate cancer findings in performed biopsies. Direct healthcare costs decreased.

**KEY POINTS**
A change from PSA to Stockholm3 for the diagnosis of prostate cancer in primary care in the Stavanger region in Norway is described and assessed.
•Implementation of a new blood-based test for prostate cancer detection in primary care was feasible. A majority of GP clinics started to use the test within three months.
•Implementation of the Stockholm3 test was followed by:
–a 28% reduction in number of men referred for urological prostate cancer work-up
–an increase in the proportion of clinically significant cancer in performed prostate biopsies from 42 to 65%
–an estimated reduction in direct health care costs between 23 and 28%.

## Introduction

Prostate cancer is the most common cancer diagnosis in Norway, and the second leading cause of cancer-related death in men. In 2017, the incidence and prevalence were 4983 and 49,722, respectively, and 934 men died of prostate cancer [1]. Traditionally, prostate-specific antigen (PSA) is used to identify men at increased risk of prostate cancer, for follow-up of men on active surveillance for low-risk prostate cancer, and for control of men after treatment for prostate cancer. For diagnostic purposes, current practice includes using PSA. However, this is associated with significant over-diagnosis and over-treatment of prostate cancer as about two-third of patients are diagnosed with clinically non-significant prostate cancer (cnsPC) defined as Gleason Score 6 [2,3]. Use of PSA is also associated with under-diagnosis and under-treatment of clinically significant prostate cancer (csPC) defined as Gleason Score ≥7 in men with low PSA [4]. Additionally, there is a need for optimized risk-stratification due to the risk of serious needle-biopsy-related complications (e.g. sepsis) as the incidence of hospitalization due to severe infections is continuously increasing [5].

Norwegian guidelines state that screening with PSA testing is recommended only for men with genetic predisposition [6]. Nevertheless, PSA testing is common. The number of conducted tests in 2011 corresponded to 45% of men above 40 years of age, and a large proportion of PSA testing of men is conducted in primary care by general practitioners (GPs) [7].

Several recently developed blood-based tests have proven superior properties compared to PSA for selecting men for prostate biopsy [8–13]. The Stockholm3 test has been developed at Karolinska Institutet in Stockholm, Sweden, and predicts a man’s risk of having a csPC in biopsy. Stockholm3 is a blood-based test including analyses of PSA and four other proteins, 101 genetic markers (single nucleotide polymorphisms) and clinical information (age, family history, earlier biopsies and use of 5-alpha reductase inhibitors) [14,15]. The result of Stockholm3 includes a recommendation for further follow-up, where men with Stockholm3 Risk Score ≥11% are recommended referral to urologist for further workup. A prospective diagnostic study including 58,818 men showed that Stockholm3 has substantially higher sensitivity and specificity for clinically significant prostate cancer than PSA [14]. The number of biopsies were reduced by 32 and 44% of the benign biopsies were avoided without compromising the sensitivity to diagnose csPC [14]. Besides technical validation, the Stockholm3 test has been externally validated in the STHLM3MRI project on a cohort of 532 Norwegian and Swedish patients already selected to perform a prostate needle-biopsy by urologists based on current practice [16,17] and in a clinical cohort of 573 men in Stockholm [18].

In 2016, after a thorough discussion involving GPs and hospital doctors, Stavanger University Hospital decided to start a project aiming to improve the diagnosis of prostate cancer. The project was launched in 2017. All GPs in the Stavanger region were recommended to change from PSA to Stockholm3 as the prostate cancer test for risk-stratification of men before referral for further urological work-up. This makes our county the first large healthcare region to replace PSA with Stockholm3 for early detection of prostate cancer in general practice.

This publication describes the early experiences of implementing a novel diagnostic test in primary care in a large healthcare region. It aims to (A) describe the GPs response to the recommendation to replace PSA with Stockholm3, (B) compare the results of replacing PSA with Stockholm3 with regard to the percentage of men being referred to biopsy, (C) compare the outcome of needle-biopsies with regard to distribution of csPC and ncsPC, and (D) estimate the impact on the direct costs of prostate cancer diagnosis.

## Design and methods

### Setting and population

The Stavanger region is a geographical area, bounded by the sea, fjords and mountains. The region has 370,000 inhabitants divided over 18 municipalities, and 304 GPs in 97 clinics, three private urologists, and one hospital. According to Statistics Norway, the number of men over the age of 40 in the region was 80,623 in 2017 and 81,953 in 2018.

### Intervention

As of September 2017, all early detection of prostate cancer in men in primary care in Stavanger region was recommended by Stavanger University Hospital to be done using Stockholm3 instead of PSA. GPs were instructed to refer to urologist for further work-up if the Stockholm3 Risk Score ≥11%, indicating an increased risk of csPS.

The implementation of Stockholm3 started with a meeting of the GPs at the Stavanger University hospital in June 2017. In August 2017 all GPs received written documentation about the new test, including detailed instructions and necessary laboratory equipment. GPs were advised to continue their diagnostic practice as before, but to use Stockholm3 instead of PSA when they decided to test patients without known prostate cancer. The start-up of the new routine was set to September 2017, and GPs were informed that after September 18th, referrals to the hospital based on PSA could be rejected because the hospital wanted to use Stockholm3 for prioritization of patients.

Since the research aims to analyse the consequences of the change from PSA to Stockholm3 in clinical practice, without affecting the demand for testing, a strategy to avoid media attention was developed in collaboration with the Norwegian Cancer Society and the Norwegian Prostate Cancer Association. Thus, information about the ongoing research was published in the general media only after the inclusion of patients in the study had ended.

The project was approved by the Regional Committee for Medical Research Ethics (2017/71 REK Vest) in March 2017.

## Method

During the implementation process, the conversion rate from PSA to Stockholm3 among the GPs was monitored. Based on the real-life outcome data from 4784 men tested (from September 1st 2017 to October 12th 2018), we compared PSA values with Stockholm3 recommendation for needle-biopsy. A cut-off for PSA ≥3ng/ml was chosen as a positive PSA test in our analysis because the Stockholm3 test was developed with a cut-off for a positive test corresponding to the risk of a csPC at PSA = 3ng/ml. In addition, this level has been used in other papers regarding the Stockholm3 test [13,14,16], and accordingly, this provides an appropriate basis for comparison.

At the department of pathology, all positive biopsy outcomes are routinely sent for registration at the Norwegian Cancer registry. These records were retrieved for the periods January to June 2017 (before the implementation of Stockholm3) and for January to June 2018 (after the implementation of Stockholm3). In cases where there was more than one report on the same patient, the report with the highest Gleason score was included in the analysis (i.e. 8 reports from 2017 and 8 reports from 2018 were excluded because the same patient was biopsied twice). Information regarding negative biopsies were not included in the analysis as negative (non-neoplastic) biopsies are not reported to the cancer registry.

## Statistical analysis

The data were analysed with descriptive statistical methods, as percentages, proportions and rates. Differences in rates were tested with chi-square tests. Results are shown with confidence intervals and *p*-values. The proportion of csPC was calculated as number of csPC divided by total number of positive biopsies. Based on cost estimates from Stavanger University Hospital combined with outcome data from 4784 men tested with Stockholm3, a simplified health economy cost-model for replacing PSA with Stockholm3 for diagnosis of prostate cancer in men was calculated. Costs were calculated as: Total costs = Cost blood sampling + cost blood analysis PSA + cost blood analysis Stockholm3 + cost trans-rectal ultrasound (TRUS) + cost magnetic resonance imaging (MRI; including MRI protocol and radiologist) + costs needle-biopsy and pathology workup + cost sepsis following needle-biopsy. Regarding costs based on PSA, separate assessment was made using a re-biopsy rate of 30% and 60%, and a post-biopsy sepsis rate of 2 and 5%. A *p*-value <0.05 was considered statistically significant.

OpenEpi (version3.01: Dean AG, Sullivan KM, Soe MM) was used to compare rates, and confidence intervals and *p*-values were calculated using their recommended methods.

## Results

### Regarding the implementation of Stockholm3

In the study period, 5005 Stockholm3 tests were conducted, of which 221 tests were excluded due to lack of documented patient consent, missing information or other errors. Thus, 4784 men underwent Stockholm3 testing and were included in the study.

**Figure 1.**
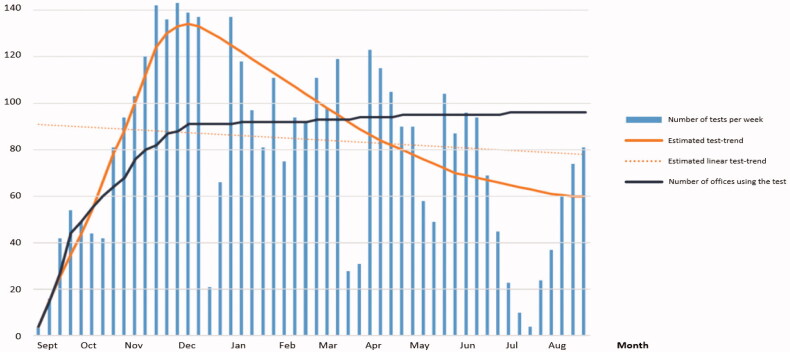
Number of Stockholm3 samples, and number of GP offices that had started to use Stockholm3, per week during the first year after initiation.

Fourteen weeks after initiation date 88 of 97 (91%) of the GP clinics in the region had at least one man conducted a Stockholm3 test, and after 12 months, only 3 of 97 (3%) GP clinics had not used the test. (Figure 1).

The test rate increased during the first three months and reached a peak of 148 tests in one week, twelve weeks after start-up. The test rate thereafter declined, showing a negative linear test trend. During national holidays (Christmas, Easter, the summer holiday), fewer men were tested (Figure 1). During the first 12 months, the mean number of Stockholm3 referrals per GP was 16, corresponding to about one test every 25th day.

PSA usage in the Stavanger region increased on average by 433 tests a year from 25,218 tests in 2010 to 27,815 tests in 2016 (before the implementation). If this trend had continued, it would have resulted in 28,249 tests in 2017 and 28,682 tests in 2018. The actual figures of PSA and Stockholm3 tests together were 27,886 in 2017 and 27,975 in 2018 (after the implementation). Accordingly, the annual increase in test activity has been reduced after the implementation of the Stockholm3 test.

### Regarding Stockholm3 compared to PSA

Of the men included in the study, 995/4784 (20.8%) had a positive Stockholm3 Risk Score (Stockholm3 Risk Score ≥11%), while 1387 (29.0%) had positive PSA (PSA ≥3.0 ng/ml) (Table 1). The proportion of cases where further referral was indicated thus differed 8 percentage points, corresponding to a 28% relative decrease in number of tested men who would be referred for further workup. In addition, 520/4784 (11%) had positive PSA and negative Stockholm3, and 128/4784 (3%) had negative PSA but positive Stockholm3 test. To mimimize unnecessary testing, men who had a Stockholm3 risk score ≤3%, 49.3% (*n* = 2358) were recommended a prolonged interval to subsequent testing [19].

**Table 1.** Distribution of 4784 Stockholm3 tests with risk score and recommendations, compared with corresponding results when based on PSA.

*Note*: Test results indicating a referral for further urological work-up is encircled.

### Regarding needle-biopsy outcome

In total, 518 needle-biopsy reports from the department of pathology regarding the diagnosed patients with prostate cancer were included in the analysis. Table 2 shows the distribution by year, age groups and Gleason score.

**Table 2.** The number and rate of positive biopsies in 2017 before the intervention (using PSA) and in 2018 after the implementation of Stockholm3, by age group and Gleason score; related to the population, and the figures for 2018 when standardizing the results regarding the population changes.

	2017 (Before implementation)				2018 (After implementation of Stockholm3)				
Age group	Population	*N*	Rate/10,000 Person years	CI	Population	*N*	Rate/10,000 Person years	CI	*p*
*Gleason 6*									
40–49	26546	2	1.5	0.3–5.0	26323	2	1.5	0.3–5.0	0.99
50–59	22599	29	25.7	17.5–36.4	23058	22	19.1	12.3–28.4	0.30
60–69	17165	68	79.2	62.0–99.8	17543	50	57.0	42.8–74.6	0.08
70–79	9796	36	73.5	52.3–100.1	10437	24	46.0	30.2–67.4	0.07
80 +	4517	0			4592	2	8.7	1.5–28.8	0.25
Sum	80623	135	33.5	28.2–39.5	81953	100	24.4	20.0–29.6	0.016
			Standardized to 2017 age-distribution				24.8	20.3–30.0	0.023
*Gleason ≥7*									
40–49	26546	0			26323	2	1.5	0.3–5.0	0.25
50–59	22599	10	8.9	4.5–15.8	23058	22	19.1	12.3–28.4	0.04
60–69	17165	34	39.6	27.9–54.7	17543	67	76.4	59.7–96.4	0.001
70–79	9796	46	93.9	69.6–124.2	10437	80	153.3	122.4–189.8	0.007
80 +	4517	8	35.4	16.5–67.3	4592	14	61.0	34.7–99.9	0.22
Sum	80623	98	24.3	19.8–29.5	81953	185	45.2	39.0–52.0	<0.000001
		Standardised to 2017 age-distribution					45.9	39.6–52.9	<0.000001

The number of csPC increased from 98 before to 185 after the implementation of the Stockholm3 test. In the same time period, the number of cnsPC decreased from 135 to 100. These changes are statistically significant, also when adjusted for the slight change in the age distribution. The proportion of biopsies positive for cancer that showed csPC increased from 42.1% (98/223) before implementation to 64.9% (185/285) after implementation of Stockholm3 in the Stavanger region. Correspondingly, both the number and the rate of cnsCP decreased from 135/57.9% before implementation to 100/35.1% after implementation of Stockholm3.

### Regarding direct costs

Using cost estimates from the Stavanger region and outcome of the study population (*n* = 4784), we calculated the impact on the direct costs of using PSA versus Stockholm3 in our study population.

According to a review [20], the percentage of negative result in first prostate biopsies when based on PSA is up to 70%. According to the European Association of Urology [21] (and also the Norwegian guidelines [6]), these patients are often recommended to be re-biopsied. We have not found documentation about level of re-biopsies in the literature, but from a population-based registry from the Stockholm region about 35% of patients biopsied on the basis of a PSA test were re-biopsied (Unpublished data, personal communication Tobias Nordström). In this assessment, we have estimated the direct costs if 35% of cases are re-biopsied when biopsies are based on PSA. When the biopsies are based on Stockholm3, we have used the registered number of re-biopsies in this study.

**Figure 2.**
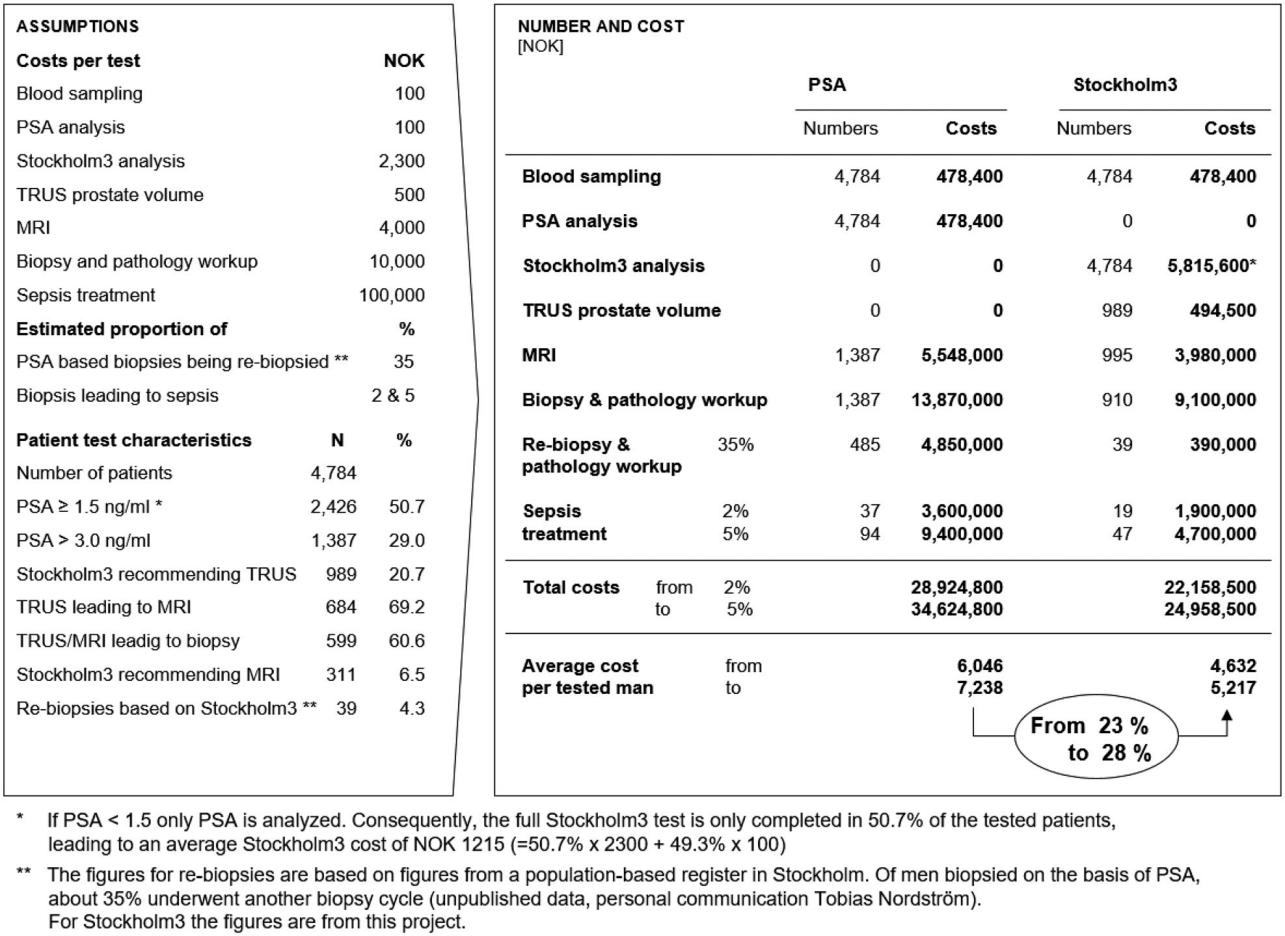
Number and costs for 4784 men, in total and per tested man, with Stockholm3 compared with estimated cost if the diagnosis would have been based on PSA.

One main effect of introducing Stockholm3 is a reduced need for biopsies. The cost of treating post-biopsy sepsis has therefore been included as a relevant cost saving directly related to the intervention. The risk of sepsis after transrectal prostate biopsy is stated to be 2–5% [22]. It is therefore included in the calculation what the costs will be if 2 or 5% of the cases got sepsis. As shown in Figure 2, the average expected cost to test one man for prostate cancer is between 6046 and 7238 Norwegian kroner using PSA ≥3 ng/ml, and between 4632 and 5217 Norwegian kroner using Stockholm3 Risk Score ≥11% as cut-off for referring a man to urologist. This corresponds to a decrease in direct healthcare costs per man tested using Stockholm3 instead of PSA of between 23 and 28% (Figure 2).

## Discussion

This study illustrates several points related to implementing a new prostate cancer diagnostic test in primary care. First, more than 90% of the GP clinics started to use the new test within 14 weeks of introducing the new procedure. On average, the GPs in the region requested the new prostate cancer test every 25th day during the study period. Secondly, using the new test instead of the traditionally used PSA, needle-biopsy referral could be reduced by 28%. Thirdly, in men undergoing biopsies, the proportion with csPC increased during the period. This indicates increased sensitivity for csPC and decreased risk of over-diagnosis. Lastly, a health-economic cost estimate showed lower costs using the new test due to lower costs for MRI, biopsies and sepsis.

One reason for the rapid implementation of the Stockholm3 might be that the GP’s were not satisfied with the use of PSA and the uncertainty regarding the interpretation of the results. Dealing with uncertainty and patients’ expectations is challenging for the GPs [7]. A Stockholm3 result includes a recommendation and not just a number. The promise of a better diagnostic test with clear guidelines is a powerful incentive for change.

As in other parts in Scandinavia, prostate cancer testing in Stavanger is common [23]. Considering the rapid implementation of the new test, it was reassuring that the annual increase in test activity in the period 2010 to 2016 was less after the implementation of Stockholm3. After the initial increase in use of Stockholm3, it appeared that the test rate declined to a new ‘steady state’ after about a year. The trend in using the test also confirms that the project managed to keep the information about the research with Stockholm3 away from the media, thus avoiding an increase in general public demand for the new test.

The 4784 Stockholm3 tests conducted during the study generated 995 biopsy recommendations. This should be compared to the 1387 biopsy recommendations resulting from using PSA ≥3 ng/ml as the deciding test. This 28% reduction in biopsy recommendations is significant not only for the men (who do not have to undergo a procedure that is laborious and potentially dangerous) but also because it frees up scarce healthcare resources in departments of urology, radiology and pathology.

It may be argued that many GPs do not necessarily refer patients with a PSA of 3 ng/ml to specialists. Instead, they may use age-related cut-off values for PSA or follow the changes in PSA over time before deciding to refer a man for further diagnosis. It is a limitation of this paper that no more detailed analysis has been made of the effect of the change from PSA to Stockholm3 for different age groups or with different cut-off values for PSA. PSA is used not only for the diagnosis of prostate cancer, but also for active surveillance of patients with low-risk prostate cancer and for the follow-up of patients being treated for prostate cancer, and it was not possible in this study to distinguish the tests taken for diagnostic purposes from the others. However, if the diagnosis of prostate cancer has previously been based on a higher PSA value, the increase in the proportion of diagnosed csPC after the implementation of the Stockholm3 test is underestimated in our analysis.

The direct cost calculation shows that introducing new, improved diagnostics in primary care does not necessary increase overall healthcare costs. Instead, the direct costs for healthcare is reduced by between 23 and 28%, because Stockholm3 reduces the numbers of MRIs, biopsies and sepsis treatment needed compared to use of PSA. It might be worth noting that the actual cost of the Stockholm3 test itself is higher than PSA, but introducing better tools for the GP may still reduce the overall costs for the healthcare system. It should also be noted that this study has not conducted a complete cost efficiency study including burden of disease impact or external costs.

There are several suggestions for novel prostate cancer tests, including 4 K Score, phi, and the Stockholm3 test [8,14]. All these tests have been shown to outperform PSA or combination of clinical information. Based on existing evidence including validation in a Scandinavian population, Stavanger Region chose to use Stockholm3 for primary prostate cancer diagnostics. Our results are in agreement with the conclusions from Capio S:t Göran Prostate Cancer Centre in Stockholm, Sweden [18] and the health authorities in Stockholm who have decided to implement the Stockholm3 test in their region [23]. It should however be noted that PSA still represents an important tool for follow-up after treatment for prostate cancer.

It is a weakness of the study that information about negative biopsies is not included in the analysis, but these figures will not affect the proportion of cnsPC versus csPC. The results of pre-biopsy MRIs is not included either, but pre-biopsy MRI have been done routinely in the region since 2013. As MRI routines have not changed during the study period, we do not attribute the change in proportion of cnsPC versus csPC to MRI. Nevertheless, the prostate cancer diagnostic process is complex and undergoing continuous development. Thus, we cannot exclude other diagnostic interventions that may have performed differently over the course of the study period, possibly affecting the differences in biopsy outcome reported here. Furthermore, PSA may still have been used during the study period, possibly diluting any measured effects related to the Stockholm3 test. Lastly, despite the isolated nature of the study site (Stavanger Region), we cannot exclude a limited number of men in Stavanger that had a prostate cancer test outside Stavanger. Likewise, men from outside the region may have sought testing and treatment in Stavanger.

## Conclusions

Replacement of PSA with Stockholm3 in primary care in the Stavanger region was feasible. Most GP clinics started using the test within three months. The need for biopsies decreased significantly while the proportion of clinically significant prostate cancer in performed biopsies increased considerably after the implementation of Stockholm3. In addition, direct healthcare costs were reduced.
